# Global Trends and Hotspots in Pediatric Anesthetic Neurotoxicity Research: A Bibliometric Analysis From 2000 to 2023

**DOI:** 10.7759/cureus.58490

**Published:** 2024-04-17

**Authors:** Xiaoqin Li, Lin Tan, Yingyi Chen, Xinyan Qin, Ze Fan

**Affiliations:** 1 Department of Anaesthesiology and Perioperative Medicine, The First Affiliated Hospital of the Air Force Medical University, Xi'an, CHN; 2 Department of Medical Ethics, College of Basic Medicine, Fourth Military Medical University, Xi'an, CHN; 3 Department of Stomatology, Xi'an Medical University, Xi'an, CHN; 4 Department of Stomatology, Xi’an Medical University, Xi'an, CHN; 5 Department of Anesthesiology, State Key Laboratory of Oral & Maxillofacial Reconstruction and Regeneration, National Clinical Research Center for Oral Diseases, Shaanxi Engineering Research, Center for Dental Materials and Advanced Manufacture, Fourth Military Medical University, Xi'an, CHN

**Keywords:** general anesthesia, neurotoxicity, publications, bibliometric analysis, research trends

## Abstract

The impact of general anesthetics on brain function development is one of the top frontier issues of public concern. However, little bibliometric analysis has investigated this territory systematically. Our study aimed to visualize the publications between 2000 and 2023 to inspire the trends and hotspots in anesthetic neurodevelopmental toxicity research. Publications from 2000 to 2023 were collected from the Web of Science Core Collection. CiteSpace was utilized to plot and analyze the network maps of countries, institutions, authors, journals, and keywords associated with these publications. A total of 864 publications, consisting of 786 original articles and 78 reviews, were extracted from 2000 to 2023. The annual publications have increased constantly over the past two decades. The USA and the People's Republic of China were the leading driving forces in this field. Harvard University was the most productive institution. Zhang Y published the most related articles, and Jevtovic-Todorovic V was mostly cited in this field. The most prolific journal was Pediatric Anesthesia, and the most frequently co-cited journal was Anesthesiology. Keywords were divided into nine clusters: "apoptosis", "propofol", "developing brain", "cognitive dysfunction", "neuronal cell degeneration", "brain", "neuroinflammation", "local anesthesia", and "oxygen therapy". The strongest citation bursts in earlier years were "learning disability", "cell death", and "cognitive function". The emerging trends in the coming years were "awake regional anesthesia", "behavioral outcome", and "infancy general anesthesia compared to spinal anesthesia". We conclude that anesthetic-induced neurotoxicity has received growing attention in the past two decades. Our findings evaluated the present status and research trends in this area, which may provide help for exploring further potential prospects on hot topics and frontiers.

## Introduction and background

Advances in modern general anesthesia have guaranteed the security of billions of surgeries annually, and many of the patients are children under three years old [1]. Accordingly, there are growing concerns about whether general anesthetics result in impairments in the developing brain of children [2-4]. Evidence from fundamental research substantiated that early repeated or long-term exposure to anesthetic agents could induce neurotoxicity, as well as functional deficits in learning, memory, and other behaviors [5, 6]. Clinical studies also revealed, though lack of definite answers, the detrimental outcomes in children's developing brains after general anesthesia [7-9]. Thus, the Food and Drug Administration (FDA) warned in 2016 that "repeated or prolonged general anesthesia in children under three years old or in fetuses in the third trimester may restrict the brain development" [10].

In the past two decades, a growing number of researchers have devoted to uncover the underlying mechanisms of anesthesia-related neurodevelopmental toxicity, and their publications are rapidly becoming invaluable sources of information for clinical use [11-14]. However, few systematic analyses of these publications have been performed, and the key milestones and emerging trends of relevant knowledge are inexplicable [15]. Therefore, it is highly important to organize the knowledge domain and explore the developmental trends of anesthesia-induced neurotoxicity [16, 17].

Bibliometrics refers to quantitative analyses for evaluating literature by using statistical and mathematical methods, which have gained wide application in many fields [18, 19]. This method enables the researchers to grasp the law of publications and predict future directions [20]. The Web of Science Core Collection (WoSCC), containing information about annual outputs, authors, instructions, and so on, is one of the most commonly used databases for bibliometrics [21]. CiteSpace is a popular tool for cooperation analyses [22]. To our knowledge, there are few bibliometric analyses on anesthesia-induced neurotoxicity. We thus aim to describe the research outputs using the abovementioned tools to determine the hotpots and development trends in this field and to provide perspectives for the investigator's future works.

In this study, we conducted a bibliometric analysis to comprehensively evaluate the research of anesthetic-induced neurotoxicity from 2000 to 2023. Full records and references of the publications were retrieved from the WoSCC. Core information about co-occurrence, co-citation, as well as the bursts were analyzed and visualized using CiteSpace. Our findings described the knowledge map of the publications in this territory, which could be helpful for informing future research and clinical applications.

## Review

Retrieval strategy

Publications on anesthetic-induced neurodevelopmental toxicity were searched in the WoSCC. The citation index includes Science Citation Index Expanded (SCI-EXPANDED), Social Sciences Citation Index (SSCI), Conference Proceedings Citation Index-Science (CPCI-S), Conference Proceedings Citation Index-Social Sciences & Humanities (CPCI-SSH), Arts & Humanities Citation Index (A&HCI), Book Citation Index-Social Sciences & Humanities (BKCI-SSH), Book Citation Index-Science (BKCI-S), Emerging Sources Citation Index (ESCI), Index Chemicus (IC) and Current Chemical Reactions Expanded (CCR-EXPANDED). The search terms were the following: (anesthesia OR anaesthesia OR anesthetic* OR anaesthetic* OR sevoflurane OR isoflurane OR desflurane OR ketamine OR propofol OR dexmedetomidine OR fentanyl OR sufentanil OR remifentanil OR etomidate OR midazolam) AND (develop* OR neurodevelop* OR fetus* OR foetus* OR fetal OR foetal OR prenatal OR antenatal OR maternal OR pregnant* OR gestation* OR postnatal OR offspring* OR newborn* OR neonate* OR infant* OR baby OR juvenile OR babies OR child* OR young OR adolescen* OR paediatric* OR pediatric*) AND (cognitive* OR behavior* OR disability OR abnormal* OR learning OR memory OR toxic* OR neurotoxic* OR neurogenesis OR neurodegeneration OR apopto* OR cell death), which were under the "advanced search" feature and the "title" category. The timespan was set from 2000-01-01 to 2023-11-08 within the publication date.

Data extraction

**Figure 1:**
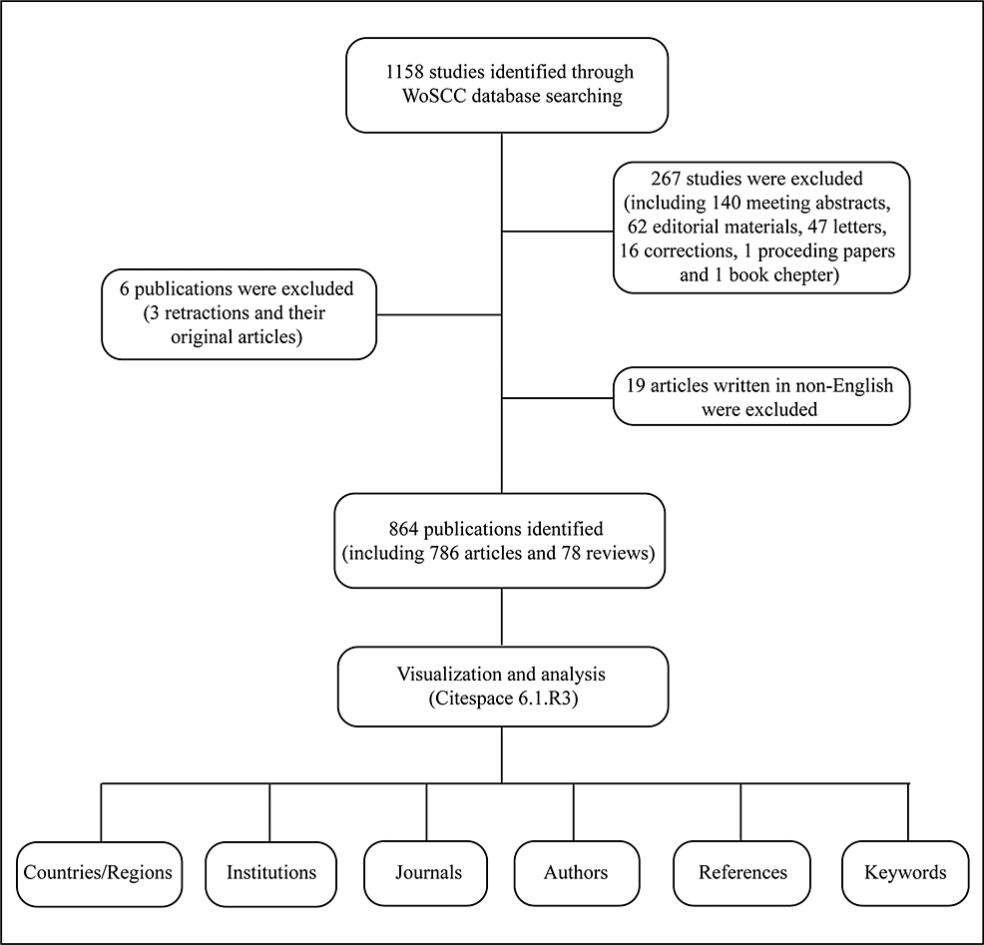
Flowchart of publication inclusion and exclusion

Following the abovementioned search strategy, a total of 1158 issues were retrieved in the WoSCC database. To guarantee the accuracy and quality of analysis, 267 issues of other source types, including meeting abstracts and letters, six retractions, and 19 non-English articles, were excluded. This query resulted in a number of 864 publications for investigation, including 786 original articles and 78 reviews (Figure 1). All results were exported to text-format files with full records and cited references.

Visualized analysis

CiteSpace software (Version 6.1.R3) was used to create the network visualization maps. Briefly, the retrieved entries were imported into the software, and the duplicate information was filtered out. The parameters were set up as follows: time slicing (from 2000 to 2023, one year per slice); node types (select one at a time); link scope (within slices); link strength (Cosine); selection criteria (top 50 items); and visualization (cluster view-static, show merged networks). Visualization knowledge maps consisted of nodes and links. The nodes represented elements of occurred items (including keyword, author, reference, etc.). The size of a node meant occurrence or citation frequency. The colors in different circles meant the years 2000 to 2023 from the center to periphery of the nodes. The purple circle of a node meant high centrality or key points in the field. The link lines among nodes meant collaborative relationships (countries, institutions, authors, etc.) or co-citation relationships (references, journals, authors, etc.). For each node type, citation count was performed for analyzing co-occurrences; Betweenness centrality was performed for evaluating the importance; Summarization of clusters was performed for searching major clusters; citation burst was performed for exploring key indicators of emerging trends. The metrics of analysis of these publications were listed as follows: trends in annual issuance, countries/regions and institutions, authors and co-cited authors, co-cited journals and references, and keywords. Further detailed information about the software can be found in Citespace manually.

Results

**Figure 2:**
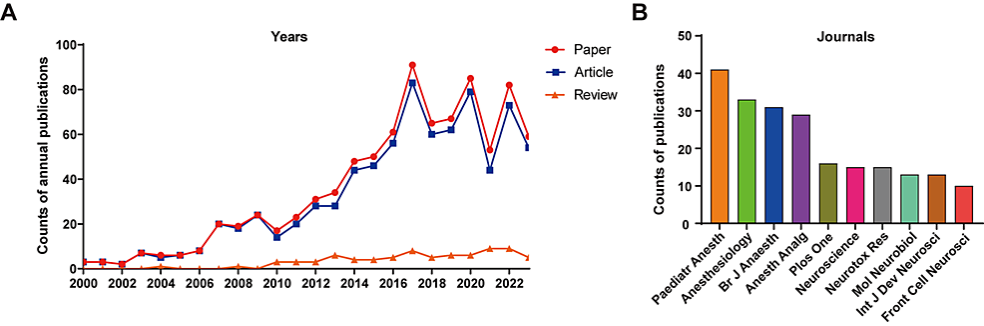
Trends of publications (A) Annual publications of papers (red), original articles (blue), and reviews (orange) from 2000 to 2023; (B) Top 10 journals with the most counts of publications related to anesthetic-induced neurotoxicity

Eight hundred sixty-four publications were collected in total, including 786 original articles and 78 reviews (Figure 1). The count of annual publications has visibly increased in the past two decades, with a steady tendency before 2006, a sharp increasing tendency between 2007 and 2017, and a fluctuation in the following six years (Figure 2A). Pediatric Anesthesia was the most prolific journal in this area, with 41 publications, followed by Anesthesiology (n=33) and British Journal of Anesthesia (n=31), as shown in Figure 2B. These results indicated that anesthetic-related neurotoxicity, as one of the top frontier issues prompted to be solved in anesthesiology, has received great attention in these years.

**Figure 3:**
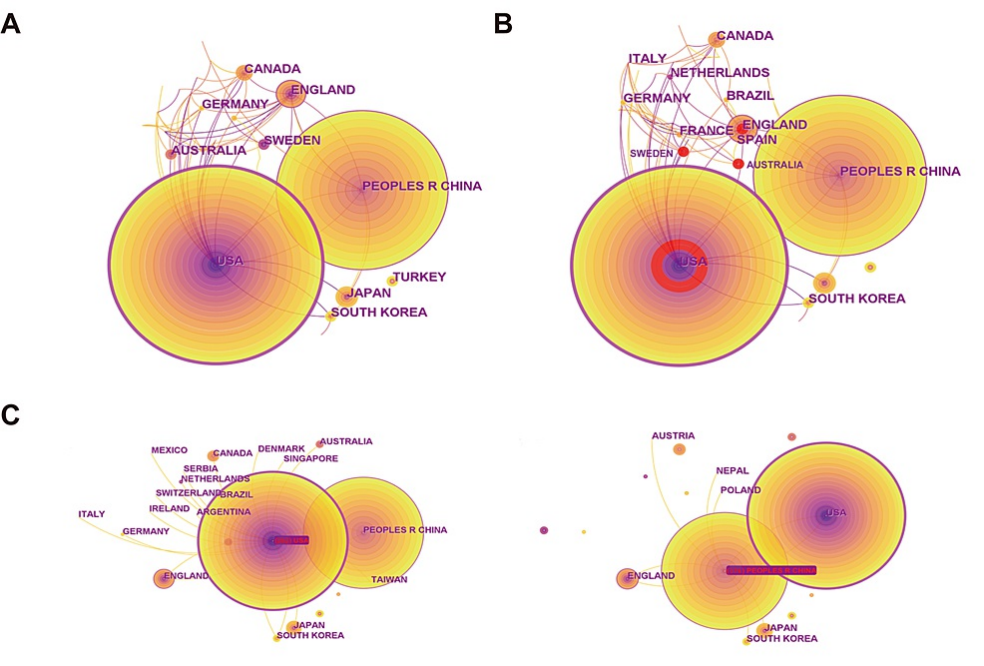
Analysis of countries/regions distribution (A) Top countries in terms of co-occurrence counts; (B) Top countries with the strongest bursts (red marks); (C) Representative collaboration networks of countries.

The map of countries and regions consisted of 50 nodes and 85 links, representing 50 countries and 85 national cooperations that contributed to the research of this field. The top 10 countries in terms of co-occurrence are displayed in Table 1. Among these countries/regions, the People's Republic of China (n=376), the USA (n=292), and England (n=39) were the top three contributors by co-occurrence, which accounted for 43.5%, 33.8%, and 4.5% of the studies, respectively (Figure 3A). Sweden, Australia, England, and the USA had the strongest citation bursts with red marks (Figure 3B). Major collaborations of the USA and People's Republic of China were displayed in Figure 3C, and the USA participated in the collaborations at most.

**Table 1:** The top 10 countries/regions of co-occurrence counts

Rank	Countries/regions	Co-occurrence
1	People's Republic of China	376
2	USA	292
3	England	39
4	Japan	33
5	Canada	33
6	Australia	29
7	South Korea	20
8	Sweden	19
9	Turkey	19
10	Brazil	18

**Figure 4:**
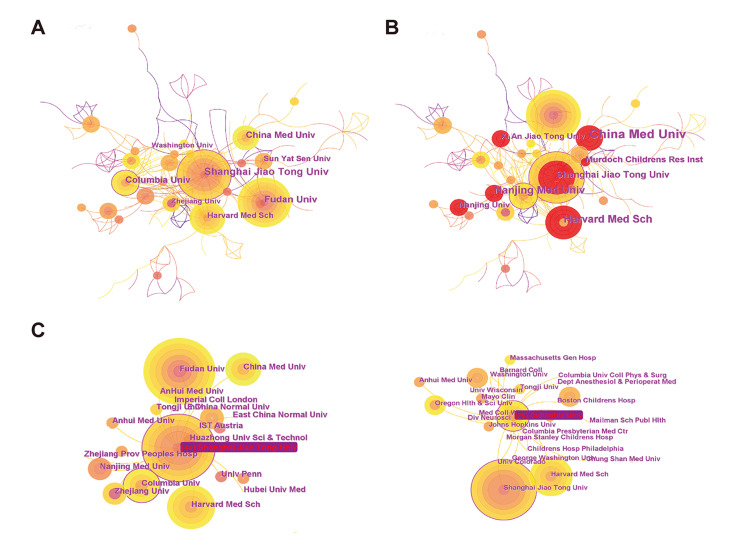
Analysis of institutions (A) Top institutions in terms of co-occurrence counts; (B) Top institutions with the strongest bursts (red marks); (C) Representative collaboration networks of partial institutions.

Papers in this field were published by 760 institutions in the cooperation network map, and the link number was 1,287. The top 10 institutions with the highest co-occurrence are shown in Table 2. The leading institution in co-occurrence analysis was Harvard University (n=57), followed by Shanghai Jiaotong University (n=44) and China Medical University (n=31) (Figure 4A). China Medical University and Harvard Medical School had the strongest bursts in recent years (Figure 4B). Shanghai Jiaotong University and Columbia University carried out the most collaborations in this field (Figure 4C). These results generally suggested that agencies from the People's Republic of China and the USA were the leading forces in this research field.

**Table 2:** The top 10 institutions of co-occurrence

Rank	Institutions	Co-occurrence
1	Harvard University	57
2	Shanghai Jiaotong University	44
3	China Medical University	31
4	Sun Yat Sen University	30
5	Fudan University	29
6	Columbia University	26
7	University of California System	24
8	Massachusetts General Hospital	20
9	Zhejiang University	19
10	Washington University	17

**Figure 5:**
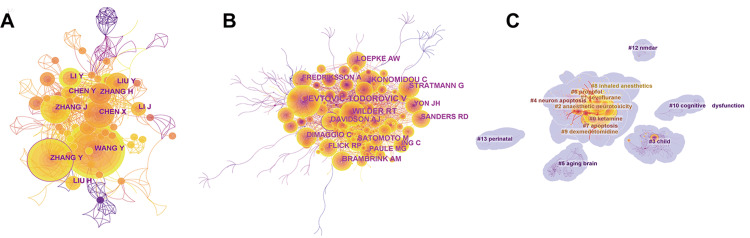
Analysis of authors and co-cited authors (A) Top authors in terms of co-occurrence counts; (B) Top authors in terms of frequency of being cited; (C) Cluster map of keywords of co-cited authors.

Five hundred ninety-seven authors and 1,381 cooperation links contributed to the outputs of this bibliometric study. Zhang Y, with the most publications (n=30), showed her influence in this field (Figure 5A). For the co-cited authors, Jevtotic-Todorovic V (frequency (freq)=304), Wilder RT (freq=201), and Ikonoidou C (freq=169) were cited most frequently (Figure 5B). The top 10 most productive and co-cited authors are shown in Table 3. The keywords of these authors could be divided into 13 clusters: "ketamine", "sevoflurane", "anaesthetic neurotoxicity", "child", "neuron apoptosis", "aging brain", "propofol", "apoptosis", "inhaled anesthetics", "dexmedetomidine", "cognitive dysfunctions", "N-methyl-D-aspartate receptor (NMDAR)", "perinatal" as shown in Figure 5C. Analyses of these authors generally revealed their contribution and authority in this research field.

**Table 3:** The top 10 authors and co-cited authors

Rank	Authors	Co-occurrence	Co-cited Authors	Co-occurrence
1	Wang Y	30	Jevtotic-Todorovic V	304
2	Zhang Y	30	Wilder RT	201
3	Liu Y	18	Ikonoidou C	169
4	Liu Y	18	Stratmann G	143
5	Chen X	18	Brambrank AM	142
6	Zhang J	18	Stomoto M	138
7	Zhang H	15	Dimaggio C	130
8	Liu H	12	Davidson AJ	128
9	Li J	12	Loepke AW	123
10	Chen Y	12	Fredriksson A	119

**Figure 6:**
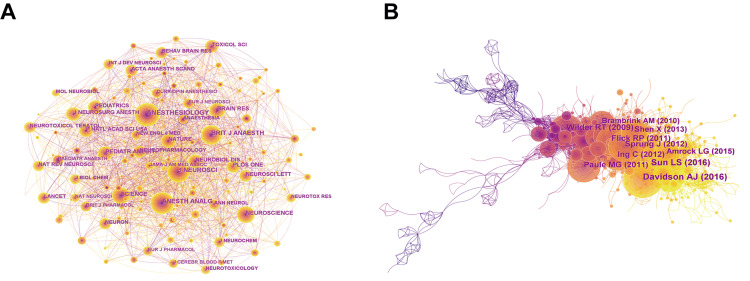
Analysis of co-cited journals and references (A) Top journals in terms of co-citation counts; (B) Top references in terms of co-citation counts.

A total of 595 nodes and 5,486 links comprised of the network map of co-cited journals. The top 10 journals with the highest co-citation are shown in Table 4. *Journal of Anesthesiology *(freq=630), *Anesthesia and Analgesia* (freq=511), and *Journal of Neuroscience* (freq=431) were the top three co-cited journals in this field (Figure 6A). The top 10 references with co-cited amounts are shown in Table 5. Davidson AJ's article published in *Lancet *(freq=81) and Sun LS's article published in *Journal of the American Medical Association (JAMA)* (freq=71) in 2016 were mostly cited, showing their popularity in this field (Figure 6B). These references provided the basis for anesthetic-induced neurotoxicity research.

**Table 4:** The top 10 co-cited journals of co-occurrence

Rank	Journals	Co-occurrence	Impact factor (2023)
1	Anesthesiology	630	8.8
2	Anesthesia & Analgesia	511	5.9
3	The Journal of Neuroscience	431	5.3
4	British Journal of Anaesthesia	406	9.8
5	Neuroscience	312	3.3
6	Science	284	56.9
7	Brain Research	279	2.9
8	PLOS One	262	3.7
9	Paediatric Anaesthesia	246	1.7
10	Pediatrics	224	8.0

**Table 5:** The top 10 references of co-occurrence

Rank	References	Co-occurrence
1	Davidson AJ (2016), Lancet	81
2	Sun LS (2016), Journal of the American Medical Association	71
3	Wilder RT (2009), Anesthesiology	60
4	Ing C (2012), Pediatrics	51
5	Flick RP (2011), Pediatrics	51
6	Sprung J (2012), Mayo Clinic Proceedings	50
7	Paule MG (2011), Neurotoxicology and Teratology	49
8	Shen X (2013), Anesthesiology	45
9	Amrock LG (2015), Anesthesiology	45
10	Brambrank AM (2010), Anesthesiology	42

**Figure 7:**
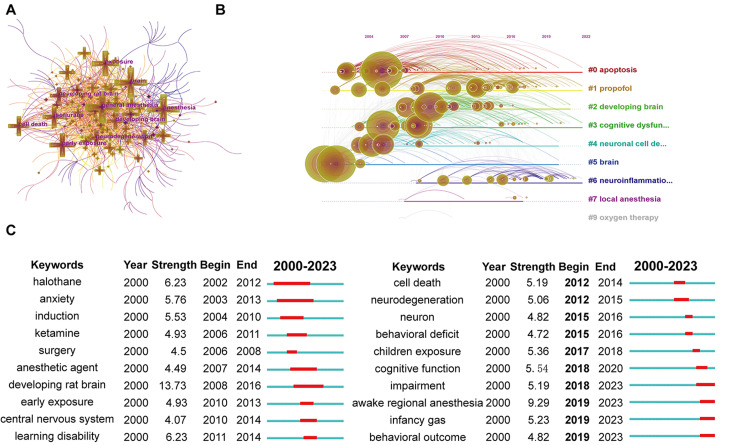
Analysis of keywords (A) Top keywords with the most co-occurrence counts; (B) Cluster map of keywords; (C) Top 20 keywords with the strongest bursts.

An increased frequency of keywords within a period could be considered an indicator for evaluating the current status or emerging trends of the research field. In total, there were 506 keywords and 3,403 links in the network map of keywords. The co-occurrence of keywords is shown in Figure 7A. Nine clusters with an overall Q=0.3912 comprised the visual knowledge map of keyword co-occurrences, including "apoptosis", "propofol", "developing brain", "cognitive dysfunction", "neuronal cell degeneration", "brain", "neuroinflammation", "local anesthesia" and "oxygen therapy" (Figure 7B). The top 20 keywords with the strongest bursts are listed in Figure 7C. Keywords such as "learning disability", "cell death", and "cognitive function" had the strongest bursts in earlier years. While "impairment", "awake regional anesthesia", "infancy general anesthesia compared to spinal anesthesia", and "behavioral outcome" were the top frontiers in burst strength in recent years, representing the hot spots and emerging trends. All these data reflected that the current understanding of anesthetic-induced neurotoxicity was significantly improved in the past two decades.

Discussion

In this article, we analyzed the knowledge domain and emerging trends in publications on anesthetic-induced neurotoxicity in the past two decades. Generally, the issues in this scientific area have received significant attention in recent years. A great number of excellent achievements have been made by many outstanding institutions worldwide, such as Shanghai Jiaotong University in China, Harvard University in the USA, and others. Keywords showed that most of the publications focused on "apoptosis" previously. In recent years, growing attention has been devoted to "neuroinflammation" and other hot spots (Figure 7B). From this point of view, interdisciplinary research and multi-institutional cooperation are essential to process this scientific field systematically.

A total of 864 publications were acquired in this study by retrieving the Web of Science Core Collection from 2000 to 2023. Actually, the problem of neurotoxicity of anesthetic drugs was raised as early as 1945 by Levy et al. [23]. In 1999, Ikonomidou et al. revealed in *Science* that injecting ketamine into rats at the peak of neurological development leads to widespread apoptosis of brain neurons, which unmasks the potential mechanism of anesthetic-induced neurotoxicity [24]. However, this research was still in its infancy before 2006 since there were no systematic reviews or clinical studies to emphasize the importance of this research in the initial years. Until 2009, it has become clear that children who received multiple anesthesia sessions prior to the age of two to four years were more likely to have cognitive dysfunction than those who received a single session of anesthesia [25]. A significant growth trend in annual publications happened in 2011 and experienced rapid development in the following 10 years. A relative symbolic event is that the FDA announced a warning in the year 2016 [10]. The annual publication outputs have slightly declined since 2018, perhaps due to the controversy and complexity of the research [4]. However, there will still be a considerable number of publications in the following years.

Analyzing sources of literature, 50 countries were involved in the research of anesthetic-induced neurotoxicity worldwide. It reflected an improvement in the safety of anesthesia management to some degree, followed by a wide interest in the long-term effect of anesthesia exposure [26]. The USA, the People's Republic of China, and England ranked high in the number of papers, which was likely due to the large population receiving general anesthesia and the advanced surgery environment in these countries [27]. Several outstanding experts, including Zhang Y from Harvard Medical School, Jevtotic-Todorovic V from the University of Colorado Anschutz Medical Campus in the USA, and Davidson AJ from Royal Children's Hospital in Australia, located in the central position with the largest number of publications as well as co-citations. The network of co-authors was distributed in a centralized manner, indicating a relatively positive cooperation among scholars in the field. More cooperation should be encouraged from different countries and institutions in the coming years.

According to the analysis of journals, the top 10 productive journals published 197 documents, accounting for 25% of the total papers. Only half of the impact factors of these publications were more than five, suggesting a difficulty in developing high-quality research in this area. However, in recent years, there have been representative articles that can reflect, to some extent, the best sources of this research field. For example, Duerden EG published an article in the comprehensive journal *Annals of Neurology* (IF (impact factor)=11.27) in 2016 [28]. Thus, we believe that there is great potential to explore and solve the scientific issue of anesthesia-related neurotoxicity in the future.

The analysis of references could reflect the hotspots of the research field to a degree. In our retrieval, the top 10 cited references were published between 2009 and 2016. These articles explored the relationship between a single anesthetic exposure before 36 months of age and the neurobehavioral outcomes in later childhood [1], the influence of ketamine use in the first week of life on long-lasting cognitive deficiency in primates [29], neonatal sevoflurane exposure as an intervention for alterations in neuronal circuits in rats [30], etc. It was noteworthy that Jevtotic-Todorovic V and Sun LS, who were also the most prolific authors in basic and clinical studies, have the most cited papers, indicating they have high academic influence and contribution in this field.

There is controversy about whether single exposures to anesthetics in infancy and early childhood affect children's neurodevelopment, and a number of retrospective studies have found that there is no necessary association between receiving a single dose of general anesthesia before the age of three years and later learning and cognitive deficits [31]. Research published in *JAMA* in 2016 suggests that receiving a single general anesthesia session before 36 months of age does not affect cognitive function and behavior around the age of 10 years [1]. A prospective study in *the Lancet* found that in young children up to 60 weeks of age who received either intrathecal anesthesia or sevoflurane inhalation anesthesia, there was no significant difference between young children at two years of age and those who did not receive anesthesia [32]. Following an FDA warning in the United States in late 2016, a retrospective study in 2017 showed that attention deficits and learning disabilities occurred at significantly higher rates in children with multiple anesthesia blips than in children without anesthesia blips [33]. Animal studies on the toxicity of anesthetic drugs have shown that the use of general anesthetic drugs during the critical period of rapid brain development or synapse formation can lead to extensive neuronal loss in developing brain tissue, as well as alterations in synaptic morphology, function, etc. However, most of the animal studies were single administrations, and the duration of anesthetic exposure in the experimental animal studies was essentially greater than one hour compared to the timeline of brain development in children, and prolonged anesthesia may interfere with the neurodevelopmental outcome of the animal animal's developing brain [34,35]. Children rarely undergo anesthesia and surgery for more than three hours unless they have congenital heart disease or special circumstances. Most of the current data comparing the toxic intensity of general anesthetics are derived from animal studies. The neurotoxicity intensity of general anesthetics also correlates with the depth of anesthesia. Surgical stimulation may affect neurocognitive function independently or there may be a synergistic effect of anesthesia on neurocognitive development [36]. Observations used in prospective studies of neurotoxicity are broadly defined as academic ability or pre-school test scores, neurodevelopment or incidence of a particular behavior, and results of neuropsychological test scales [4]. All three types of outcome indicators suffer from a lack of objectivity and sensitivity: firstly, inconsistencies in conditions or environments other than the intervening factors in retrospective cohort studies may bias the results; secondly, the clinical study included cases with a long duration from enrollment to the end of follow-up, making it difficult to track the timing and dosage of medications used during anesthesia in the patients; thirdly, due to the lack of recognized guidelines or gold standards, the primary and secondary observations used in clinical research vary. Finally, anesthesia techniques are constantly advancing, the types, dosages, and timing of anesthetics are being updated, and the patients included in the retrospective study were not from the same era. This may result in differences in outcome indicators [37].

We used several indexes of keywords, including frequency, clusters, and bursts, to reflect and forecast the research hotspots in this field. These hotspots could be summarized as nine clusters: "apoptosis", "propofol", "developing brain", "cognitive dysfunction", "neuronal cell degeneration", "brain", "neuroinflammation", "local anesthesia", and "oxygen therapy". Among these keywords, previous studies mainly focused on "anxiety", "learning disability", "cell death", and so on. The emerging trends in the coming years were "impairment", "awake regional anesthesia", "behavioral outcome", "infancy general anesthesia compared to spinal anesthesia", etc. New directions could be found in these keywords for further development of this research field.

There were several limitations in this study. Firstly, we took "Title" as the search category to analyze, but some researchers may put important terms into "keywords" rather than "title". This may cause bias in retrieved publications. Secondly, only the WoSCC database was retrieved for articles, and only issues published in English were included. As anesthetic-induced neurotoxicity also received attention in China, more databases (e.g., China National Knowledge Infrastructure (CNKI)) and more non-English papers could be included to present a broader overview of this scientific area in the future.

## Conclusions

In conclusion, this bibliometric study provides information on the emerging trends and knowledge domain in the research of anesthetic-induced neurotoxicity and aims to identify new directions and hot topics for future research in this territory. Based on previous studies, the main thrust of the study continues to emphasize the need to be alert to the effects of anesthetics on the neurodevelopment of young children or fetuses. Therefore, there is an urgent need for animal studies that mimic the clinical setting to explore in depth the mechanisms of neurotoxicity in repeated exposure to anesthetics, probing the susceptibility period for neurotoxicity of general anesthetics in the developing brain. We believe that with the development of modern medical technology and the clarification of anesthesia mechanisms, the adverse effects of anesthesia will be reduced to a minimum.
